# Ozone induced structural variation in OSA waxy rice starch: Effects on the thermal behavior of starch and its stabilized pickering emulsion

**DOI:** 10.1016/j.fochx.2024.101701

**Published:** 2024-07-27

**Authors:** Meng Du, Lei Chen, Zia-ud Din, Xinya Liu, Xi Chen, Yuehui Wang, Kun Zhuang, Lijie Zhu, Wenping Ding

**Affiliations:** a Key Laboratory for Deep Processing of Major Grain and Oil, Ministry of Education, Hubei Key Laboratory for Processing and Transformation of Agricultural Products, Wuhan Polytechnic University, Wuhan 430023, PR China; b School of Food Science and Engineering, Wuhan Polytechnic University, Wuhan 430023, PR China; c Department of Microbiology and Biotechnology, Atta ur Rahman School of Applied Biosciences (ASAB), National University of Sciences and Technology (NUST), H-12, Islamabad 44000, Pakistan

**Keywords:** Ozone, OSA-starch, Oxidation, Thermal properties, Emulsion

## Abstract

Waxy rice starch (St) was modified by pre-OSA esterification reaction followed by ozone treatment. The molecular structure of this modified product (OSA-OSt) was characterized, and the thermal behaviors and its stabilized Pickering emulsion were evaluated. ^1^HNMR and XPS results discovered that ozone initially oxidized the hydroxyl groups in the amorphous region of starch (preferentially C2/C3) along with a degree of crosslinking, enhancing the molecular orderliness. This led to an increase in water-holding capability (29.15%) and swelling power (52.8 g/g), and a decrease in solubility (0.35%). TGA, RVA, and DSC indicated that oxidation-induced crosslinking within a brief treatment period enhanced the starch's thermal stability. The structural change enabled the formation of a weak gel structure during the heating process, which displayed high thermal and freeze-thaw stability. The work proves ozone is an effective way of improving the thermal behavior of OSA-starch and its emulsion for subsequent applications in numerous food products.

## Highlights

• Ozone initially oxidized the hydroxy group (preferably at C2/C3).
• The swelling powder of OSA-OSt is enhanced by negative charge and depolymerization.
• The water solubility of OSA-OSt is constrained by the cross-linking structure induced by ozone.
• The thermal stability of OSA-OSt is improved by a cross-linking structure induced by ozone.
• The heating process resulted in the formation of a fragile gel-like emulsion with adequate thermal stability.

## Graphical abstract

**Figure:**
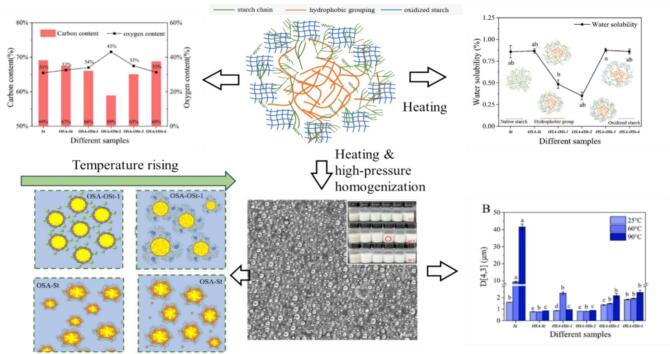

## 1 Introduction

Waxy rice starch (St) has a high concentration of amylopectin (98%). It is high adhesiveness and great resistance to freezing and thawing make it popular in enhancing the stability of various starch-based materials, such as package, emulsion, etc. (Chang et al., 1997; Kasprzak et al., 2019; Klaochanpong, Puncha-Arnon, Uttapap, Puttanlek, & Rungsardthong, 2017; Phan, Debeaufort, Luu, & Voilley, 2005). Besides, owing to the relatively higher crystallinity (around 37.18%) and smaller particle size (around 5–15 μm) compared to other types of waxy starch, St exhibits rapid gelatinization and high modifying efficiency(Hsieh, Liu, Whaley, & Shi, 2019; Simonin, Guyon, Orlowska, de Lamballerie, & Le-Bail, 2011; Vandeputte, Vermeylen, Geeroms, & Delcour, 2003; Yulianingsih & Gohtani, 2019). Recently, it was observed that the gelatinized St displayed a great dispersion in the aqueous phase, and was considered as an ideal material for architecting delivery systems due to its ability to form a colloidal gel matrix with superior retardant (Laovachirasuwan, Peerapattana, Srijesdaruk, Chitropas, & Otsuka, 2010; Yulianingsih & Gohtani, 2019). However, St still has several limitations regarding its emulsion such as the high hydrophilicity and structural vulnerability under thermal conditions (Butt, Ali, & Hasnain, 2019; Laovachirasuwan et al., 2010).

The esterification of Octenyl succinic anhydride (OSA) is a popular method for introducing hydrophobic elements into starch composition to modify the surface characteristics (Lv et al., 2018). According to the previous literature, the ability of OSA starch to emulsify is largely dependent on the amount of substitution (DS) (Feng, Zhang, Fu, & Huang, 2022). In addition to the hydrophobic interaction, the underlying mechanism of OSA starch stabilizing the emulsion could likely be attributed to the compact layer formed by the highly branched polymers and electric repulsion forces (Mu, Karthik, Chen, Holmes, & Ettelaie, 2021; Wang et al., 2023). Remarkably, the protonation of the OSA-starch-based emulsion system under lower DS limits the impact of the electric interaction, which is one of the most important noncovalent interactions in emulsion (Feng et al., 2022; Li, Chen, & Zhu, 2023). Considering the low reaction efficiency of OSA esterification and the positive impact of excessive DS on the oil/water interface, it was thus hypothesized that the additional incorporation of electric charges into the OSA starch could potentially enhance the starch's stability to a further extent at the interface between oil and water.

Ozone oxidization is a powerful technique for altering the surface structure of starch, whereas traditional oxidation is susceptible to potential safety issues. Compared to conventional oxidizing agents, ozone gas receives extensive attention due to its approved safety, simple operation, and low solvent wastage (Hu et al., 2022). Several studies investigated the effect of ozone on starch structure, where hydroxyl groups are converted to carboxyl and carbonyl groups through oxidation, and then starch molecules are depolymerized by breaking glycosidic bonds. This will cause the surface pores of starch granules to become deeper and wider, which significantly changes the surface structure of starch. (Hu et al., 2022; M. Zhang et al., 2023). In addition, ozone treatment would also simultaneously increase the number of extended chains on the starch surface.(H.-T. Chan et al., 2011; H. T. Chan, Bhat, & Karim, 2009; Castanha, da Matta and Augusto, 2017, Handarini, Hamdani, Cahyana and Setiasih, 2020) In the previous study, a post-treatment of OSA starch by ozone was performed to develop a novel starch derivative, and this dual-modified starch-based emulsion showed an increased zeta potential (Du et al., 2023). However, as starch gelatinization is critical in the process of emulsion preparation, evaluating the thermal behavior of OSA starch and its Pickering emulsion is necessary and important for revealing the inner structure of this dual-modified starch-based emulsion.

The objectives of this study encompassed the examination of the chemical structural variation of this dual modified St with OSA and ozone treatment through Fourier Transform infrared spectroscopy (FTIR), X-ray diffraction(XRD), X-ray photoelectron spectroscopy (XPS) and ^1^H-Nuclear magnetic resonance (^1^H NMR), as well as the exploration of the thermal characteristics of the modified starch and its emulsion. This study offers a crucial strategy for creating starch emulsifiers with superior emulsifying capabilities.

## 2 Materials and methods

### 2.1 Materials

Waxy rice starch (St) was provided by J Jiangsu Baobao Suqian National Biotechnology CO., Ltd. (Jiangsu, China). Shanghai Yuanye Biotechnology Co., Ltd. was responsible for acquiring Octenyl succinic anhydride (OSA, 99% purity) (Shanghai, China). The local supermarket provided soybean oil of food-grade composition, with a density of 0.917 g/mL. The remaining chemicals met the standards of analytical grade.

### 2.2 OSA starch preparation

The OSA modification of starch was done by following the method of previous work with minor modifications (Li, Chen, & Zhu, 2023) (Y. Zhang et al., 2020). The St is suspended in Milli-Q-water water (35%, *w*/*v*). Octenyl succinic anhydride (3.0% of the weight of St) was slowly poured onto the starch suspension with continuous stirring thoroughly using a magnetic stirrer and a controlled temperature of 35 °C. The pH level was maintained at 8.6 by introducing 3.0% (*w*/*v*) NaOH solution while stirring. The pH level of the solution was adjusted to 6.5 using a 3% hydrochloric acid solution (w/v) to halt the reaction. Then, the items were rinsed with 75% ethanol and water on three separate occasions. Subsequently, the sediment was subjected to drying in a 45 °C oven, followed by crushing and subsequent sieving through a 100 mesh sieve. The sample's degree of substitution was determined using the methodology suggested by (Hui, Qi-he, Ming-liang, Qiong, & Guo-qing, 2009). The DS of the sample was 0.0144.

### 2.3 Preparation of oxidized starch

The Buchner funnel was filled with OSA-St and covered with four layers of skimmed cotton gauze. Eight layers of defatted gauze were used to seal the Buchner funnel head, which was then secured with cotton thread (Li et al., 2023). The outlet of the filter flask was linked to the ozone generator. Adjustments were made to the ozone flow rate to reach 1 L/min. The preparation of OSA-St samples involved altering their oxidation durations (0.5, 1, 2, and 3 h), resulting in the names OSA-OSt-1, OSA-OSt-2, OSA-OSt-3, and OSA-OSt-4, respectively. The samples were left in an unsealed sample bag for a period of 12 h to eliminate any surplus ozone.

### 2.4 Determination of solubility, swelling power, and water-holding capacity

The solubility, swelling power, and water-holding capacity were evaluated as described by Fonseca et al. (Fonseca et al., 2015) with some modifications. St, OSA-St, and OSA-OSt were respectively used to prepare 1% (*w*/*v*) starch suspension, heated in a Shock water bath at 90 °C for 30 min, then cooled to 25 °C, and centrifuged (4000 rpm, 30 min). The supernatant fluid was placed in a constant-weight aluminum box and dried at 105 °C for 6–10 h until the weight did not change. The solubility, swelling power, and water-holding capacity of St, OSA-St, and OSA-OSt were calculated as follows:

(1) $$S=\frac{W_{2}}{W_{1}}\times 100\%$$

(2) $$B=\frac{W_{3}}{W_{1}\left(1-S\right)}\;$$

(3) $$C=\frac{W_{3}}{W_{1}}\;$$

where: S is solubility/%; B is swelling power/%; C is the water-holding capacity; W_1_ is the dry weight of sample/g; W_2_ represents the dry weight/g of starch in the supernatant fluid; W_3_ represents the wet weight/g of starch precipitated in the bottom layer.

### 2.5 Fourier transform infrared spectroscopy (FTIR) and X-ray diffraction(XRD)

Firstly, balance the starch in an oven at 40 °C for 12 h, and mixed the starch powder with KBr (1:100, *w*/w). After grinding, place it in a vacuum compressor and quickly press it into sheets. The FTIR spectra of the samples were detected with a Fourier transform infrared spectrometer (IRTracer-100, JPN) in a band of wave numbers in the range of 4000–400 cm^−1^at a resolution of 4°cm^−1^ with 32 scans with pure potassium bromide sheet as the background correction sample. All spectra were depicted using Origin 2021. Every spectrum underwent baseline correction and deconvolution through the application of OMNIC9.2 spectral analysis software (Riyajan & Poolyarat, 2023).

X-ray diffractograms for samples were captured at various intervals using a powder X-ray diffractometer (Rigaku SmartLab SE, JPN), employing Cu-Kα radiation within an angular span of 2θ 5–50°, operating at 40KV and 100 mA, and then at a scanning velocity of 5°/min with a 0.02 step increment. Jade software was utilized to fit and analyze the overall relative crystallinity.(He, Ye, Tao, Zhang, & Zhao, 2023).

### 2.6 X-ray photoelectron spectroscopy (XPS)

By using X-ray photoelectron spectroscopy, the surface chemical composition of sample can be analyzed (Thermo Fischer, ESCALAB Xi, USA). The working voltage is 12.5 kV and the filament current is 16 mA. Gather the signal following the conditions of the sample. Traditional scanning allows for the acquisition of the spectrum at a passing energy of 100 eV with a resolution of 1.0 eV, while the high-resolution spectrum is obtained at a passing energy of 20 eV with a resolution of 0.05 eV. The XPS spectrum was corrected with C1s = 284.80 eV Binding energy as the energy standard. Advantage software is used to process data.

### 2.7 ^1^H-nuclear magnetic resonance characterization (^1^H NMR)

Sample (20 mg) in 0.6 mL Dimethyl sulfoxide solution (DMSO‑*d*_6_, based on 0.03% tetramethyl silane (TMS) (0 ppm)), ultrasonic for 1 h in a nuclear magnetic tube, and then vibrate for 1 h in 55 °C water bath. Record the spectral spectrometer on Bruker 600 NMR (Bruker company, Germany). Analysis of ^1^HNMR data was done using Mestrenova software (Boetje et al., 2023).

### 2.8 Thermal gravimetric analysis (TGA)

Thermal gravimetric analysis (TGA) tests were carried out using a thermogravimetric analyzer (TGA, Mettler Toledo, Switzerland). The degradation process was measured in the range of 25–600 °C using a thermogravimetric analysis system under 20 mL/min nitrogen flow at a heating rate of 10 °C/min.

### 2.9 Pasting properties

The pasting properties of samples were determined by a Rapid Viscosity Analyzer (Super 4, Newport Scientific Instrument, Sweden). Disperse the powder sample (3 g) among the deionized water (25 mL). The RVA test is automatically terminated after the measurement cycle. The test procedure was set as follows: 50 °C holding for 2 min, heating to 95 °C (12 °C/min), 95 °C balancing for 2.5 min, then dropping to 50 °C (12 °C/min), 50 °C balancing for 2 min (Castanha, da Matta, & Augusto, 2017).

### 2.10 Differential scanning calorimetry (DSC)

The determination of the DSC was carried out per the methodology employed by Ge et al. (Ge et al., 2023). The differential scanning calorimeter was used to assess the thermal characteristics of the samples (DSC) (Q2000, TA, USA). Distilled water (10 μL) and the sample (3 mg) were placed into a closed aluminum pan. We used the empty pan as a baseline. Samples were then stored overnight at room temperature. The test temperature increased from 25 °C to 110 °C at a rate of 5 °C/min in the nitrogen atmosphere.

### 2.11 Preparation of emulsions

A mixture of St, OSA-St, and OSA-OSt (3%, *w*/*v*) was placed in distilled water and heated to 90 °C with stirring for 15 min. The starch solution (21 g) was then blended with soybean oil (9 g) using a high-speed homogenizer (XHY-DF, Xinzhi Biotechnology, China) at 10000 rpm for 2 min. Subsequently, a high-pressure homogenizer (ATS, Canada) was maintained at 20 MPa for three cycles.

### 2.12 Optical microscope and confocal laser scanning microscope (CLSM)

The microscope structure of the emulsion was taken with the optical microscope (CX40, Shunyu Optical Technology Co., Ltd., China). The microscopic structure of the emulsion was photographed with an optical microscope. Take 50 μL emulsion and add it to 5 mL deionized water for dilution, then take out 20 μL dilution drop and add it to the slide, covering the cover slide. The microstructure of the emulsion was recorded using an optical microscope, with objective multiples of 40 x.

A confocal laser scanning microscope was utilized to examine the morphology of the emulsion and interface structure. (CLSM) (OLYMPUS FV1200, Olympus, Japan). The Nile Blue solution, derived from the dissolution of Nile Blue in distilled water, was utilized to label the starch particles. The oil phase was labeled by employing a Nile Red indicator, which was derived from dissolving the Nile Red powder in anhydrous ethanol. 1 mL of emulsions was mixed with 20 μL of Nile Blue and Nile Red for 1 min. The selected excitation wavelengths were 561 nm and 633 nm.

### 2.13 Thermal stability of the emulsion

Fresh emulsions underwent heating at 60 °C and 90 °C for 15 min, followed by measuring the particle size of these emulsions using a Mastersizer 3000 (Mastersizer 3000, Malvern Instruments Ltd., UK). The emulsion was allowed to move down into the sample cell, stirring at 2000 rpm and maintaining a 10% refraction rate. Shading indices of 1.46 and 1.33 were used to depict the oil and water phases, respectively. The tests were carried out in triplicates.

### 2.14 Freeze-thaw stability of the emulsion

The emulsion was placed in the environment of −18 °C for 24 h, and then all samples were taken out and thawed in a 30 °C water bath for 2 h, and then the particle size of the sample was determined by a Mastersizer (Mastersizer 3000, Malvern Instruments Ltd., UK). The test conditions are equivalent to Section 2.13. All tests were performed three times.

### 2.15 Dynamical rheological behavior of emulsion

The emulsion's rheological characteristics are quantified using a rheometer (AR 1500, TA Instruments, United Kingdom). Increasing the temperature from 25 °C to 90 °C at a rate of 5 °C/min, and using silicone oil to seal the sample during the measurement process to prevent evaporation. Choose a fixture with a 40 mm diameter. Recorded were the variations in the storage modulus (G'), the loss modulus (G"), and the tanδ value. (Castanha et al., 2020).

### 2.16 Statistical analysis

The SPSS software package (SPSS 18.0 for Windows, SPSS Inc., USA) was utilized to conduct statistical analysis through ANOVA. Tukey's test to detect mean differences, with *p* < 0.05 for the difference was statistically significant.

## 3 Results and discussion

### 3.1 Apparent properties

**Fig. 1:**
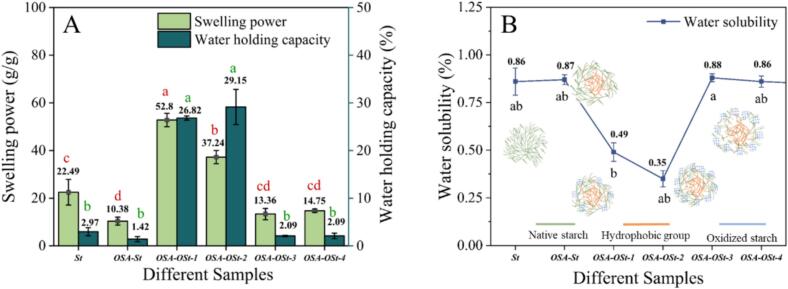
The swelling power and water holding capacity (A), and water solubility (B) of St, OSA-St, and OSA-OSt with different oxidation degrees.

The swelling power (SP), water holding capacity (WHC), and water solubility (WS) were measured to investigate the molecular state of starch samples during the heating procedure and the results are shown in Fig. 1.

From Fig. 1 A, it was noted that OSA esterification reduced the SP of waxy rice starch (St), and this result was different from the previous reports where the OSA-starch displayed an increasing SP (Ovando-Martinez, Whitney, Ozsisli, & Simsek, 2017; Siroha, Bangar, Sandhu, Lorenzo, & Trif, 2022). The results might be ascribed to the reinforced connections among branched molecules in starch particles, due to the tight binding of the double helix structure, complicating the dissolution of starch molecules or altering their internal composition. The dynamics between crystalline and amorphous areas change, hindering the dissolution of starch molecules from the particles. (Shin, Kim, Ha, Lee, & Moon, 2005). As the oxidation time increased, the OSA-OSt swelling power initially increased (OSA-OSt-1,2) and then decreased (OSA-OSt-3,4). The increase in swelling power (OSA-OSt-1,2) might be due to oxidation and degradation reactions occurring in the amorphous region, leading to the disintegration of the region, leaching of small molecule starch chains, and increasing the swelling degree of starch particles (Hu et al., 2022). While the decrease (OSA-OSt-3, 4) in the swelling power could be due to the cross-linking reaction-induced agglomeration between the leaching of small molecule starch chains and the branching degree decreased (Huang, Chen, & Liu, 2024) (Castanha, da Matta, & Augusto, 2017) (Sandhu, Manthey, & Simsek, 2012). As can be seen from Fig. 1 A, a slight decrease (*p* < 0.05) in WHC was observed for OSA-St as compared to St sample, which might be due to the increased hydrophobicity of the alkenyl long chain in the OSA residues. Remarkably, OSA-OSt-2 displayed the highest WHC. The higher WHC is associated with the increasing width of molecular spacing among starch granules and the formation of functional groups (aldehydes or carboxyls) during the oxidation process. Therefore, the repulsive interactions between oxidized starch groups may promote the entry of water into starch particles, leading to higher WHC (Pranoto, Paramita, Cahyanto, & Benjakul, 2021). The WHC of OSA-OSt-3,4 was decreased which may be related to the disintegration of starch structure. Similar findings regarding the results were observed by Oladebeye, Oshodi, Amoo, and Abd Karim (2013) in their work on ozone-oxidized cocoyam and yam starches. The water solubility (WS) of St, OSA-St, and OSA-OSt with different oxidation degrees is shown in Fig. 1B. Compared with St, the water solubility (WS) of OSA-St had no significant changes. A significant reduction in OSA-OSt solubility was observed (OSA-OSt-1,2), which was attributed to the crosslinking effect of the leaching or extension in oxidized starch, which hindered the leaching of amylopectin. Remarkably, as the ozone time was increased beyond 1 h, the WS of OSA-OSt-3 increased. The reason could be the accelerated leaching or lengthening of small molecule oxidized starch, coupled with heightened electrostatic repulsion among charges, which complicates intermolecular binding and enhances solubility. (H. T. Chan et al., 2009) (Castanha, da Matta, & Augusto, 2017) (Castanha, Santos, Cunha, & Augusto, 2019).

### 3.2 Fourier transform infrared spectroscopy (FTIR) and X-ray diffraction(XRD)

**Fig. 2:**
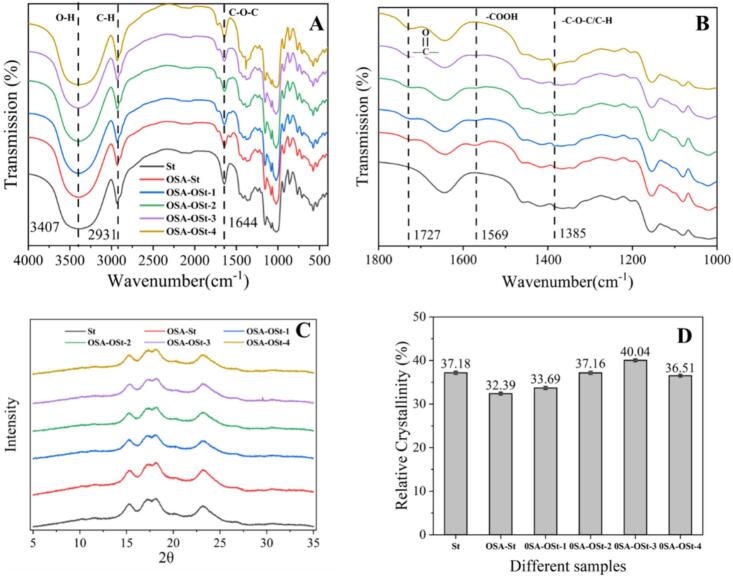
Original (A) and deconvoluted (B) FTIR spectra of different samples; X-ray diffraction patterns (C) and relative crystallinity (RC) (D) of different samples.

The FTIR spectra of native and modified starch are shown in Fig. 2 A. The stretching vibration of O—H generates an infrared absorption band between 3500 and 3000 cm^−1^(Huang et al., 2024). The primary cause of the 2931 cm^−1^ peak is the C—H stretching vibration within the α-D- (+)-dehydrated glucose unit. The observed peak in transmission at 1644 cm^−1^ is ascribed to the bending oscillation of water absorption occurring in the starch's amorphous area.(Klein et al., 2014). Regions between 600 and 1500 cm^−1^ are referred to as the fingerprint(Teixeira & del Mastro, 2023). Compared with St, the FTIR spectra of OSA-St were observed at 1727 and 1569 cm^−1^ to two new peaks (Fig. 2B) (Wang et al., 2022). The peak is related to the stretching vibration of the ester group at 1727 cm^−1^, while 1569 cm^−1^ is attributed to the asymmetry of the carboxyl group introduced by the OSA group vibration. After the ozone oxidation of OSA starch (OSA-OSt), the peak at 1569 cm^−1^ gradually disappeared, and a new characteristic peak appeared at 1385 cm^−1^. The observed peak near 1385 cm^−1^ aligns with the hydroxyl stretch vibration of the carboxyl group, a result of ozone breakdown or the creation of hemiacetal from the resulting carbonyl compound (aldehyde).

Amylose and short-chain fractions in amylopectin form an ordered double helix structure, which is a short-range ordered structure. The short-range structure of starch is sensitive to the changes of infrared spectroscopy at the molecular level. It is believed that the 1050 cm^−1^ absorbance band is responsive to structured or crystalline formations, while the 1021 cm^−1^ band is linked to an amorphous arrangement in starch. (van Soest, Tournois, de Wit, & Vliegenthart, 1995) (Ge, Shi, Wu, Wei, & Cao, 2023). Thus, the ratio of intensity of 1050/1021 cm^−1^ can express the degree of order (DO) in starch. The degree of double helix (DD) was recognized by calculating the ratio of that of absorption bands at 1021/995 cm^−1^. The 1021/995 cm^−1^ and 1050/1021 cm^−1^ ratios are shown in Table S1. The St showed the highest DO values. After OSA treatment, the DO values of all other samples were decreased. A similar trend in the relative crystallinity of XRD analysis was observed, the ratio of 1047 to 1022 cm^−1^ was initially diminished and subsequently increased as ozone duration was extended, signifying that oxidation altered the starch chain configurations, resulting in more shapeless formations.(Klein et al., 2014). The lowest DO value was exhibited by OSA-OSt-4 and subsequently exhibited the highest DD value. This finding supported the assumption that the moderate ozone treatment favored the formation of a relatively ordered crystal structure, which might be due to the cross-linking reaction between the aldehyde group and the hydroxyl group of the adjacent starch molecules stabilizing starch structure (Fig.S1) (Hu et al., 2022).

The X-ray diffraction patterns and relative crystallinity (RC) of St, OSA-St, and OSA-OSt (1–4) are shown in Fig. 2 (C&D). OSA-St and OSA-OSt exhibited X-ray diffraction patterns similar to St. Each sample's diffraction pattern showed primary peaks at angles of 15°, 17°, 18°, and 23° (Fig. 2C), which exhibited an A-type starch crystalline structure. The results suggested that neither esterification nor ozone OSA modify the crystalline form of starch. The RC is shown in Fig. 2D, implying that the OSA modification led to a decrease in the RC of the samples, decreasing from 37.18% in the native form to 32.39% in the OSA-modified St (Shweta Kumar & Saxena, 2021). After ozone treatment, the RC of OSA-OSt-1-3 increased, while it slightly decreased in OSA-OSt-4. J. W. Hu et al. (Hu et al., 2022) suggested that the generation of aldehyde groups and the formation of hemiacetal cross-linking exceed the influence of slight depolymerization during low-level oxidation. When subjected to long-term ozone treatment, the crystalline flakes of oxidized starch begin to further oxidize which explains the reason for the decrease in RC compared to medium and short ozone treatment.

### 3.3 X-ray photoelectron spectroscopy (XPS)

**Fig. 3:**
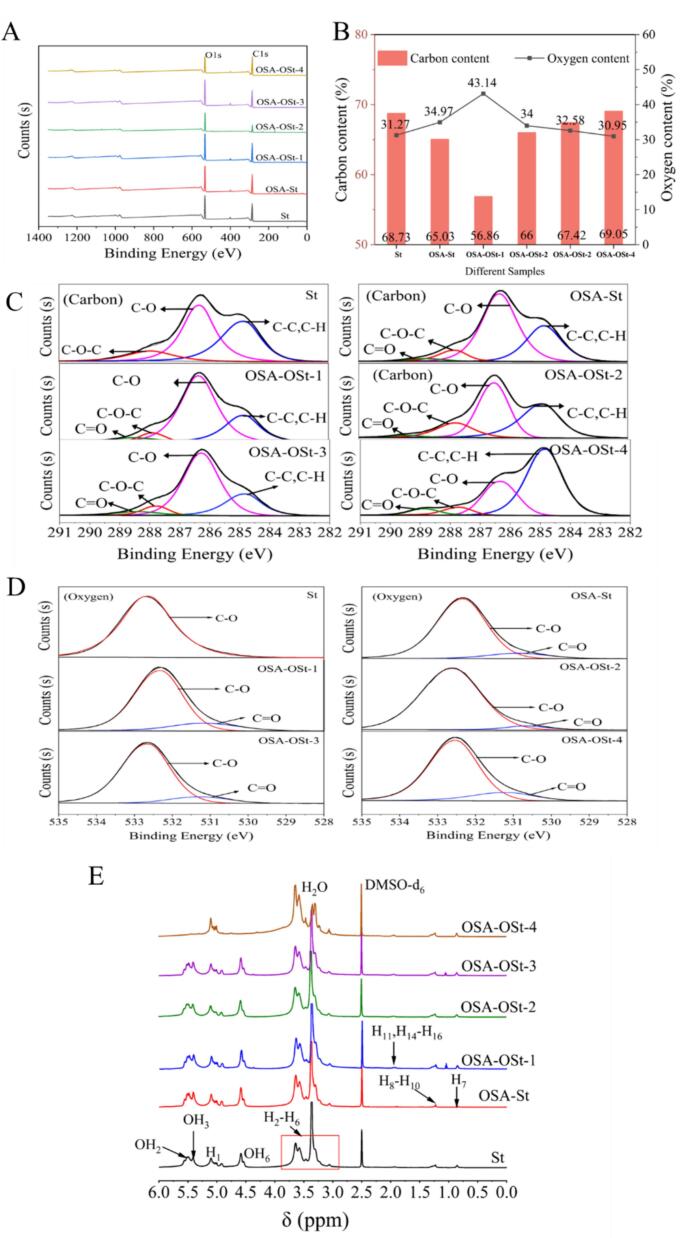
The general XPS (A) spectra of St, OSA-St, and OSA-OSt with different oxidation degrees; The C1S elemental energy(B) spectra of all samples, and the O1s narrow spectrum (D) of all samples. ^1^H NMR spectra (E) of St, OSA-St, and OSA-OSt with different oxidation degrees.

The XPS was obtained to further verify the structure of samples by measuring the chemical environment of carbon (C) and oxygen (O) atoms (Fig. 3). Fig. 3A illustrates the full-scan XPS spectra of St, OSA-St, and OSA-Ost, which include predominantly C and O components.

The C and O elements of St accounted for 68.73 and 31.27%, respectively. After esterification, the C and O elements of OSA-St were changed to 65.03 and 34.97%. In comparison to OSA-St, OSA-OSt-2 contained the maximum content of oxygen elements, suggesting the saturate introduction of oxygen atoms that occurred primarily during the hydroxyl oxidation reaction when a molecular breakdown did not play a dominant role. The XPS spectrum of C1s of St, OSA-St, and OSA-OSt is shown in Fig. 3C. The C1s spectrum of St could be illustrated by three characteristics peaks: the C—C and C—H characteristic peak (binding energy 284.8 eV), the C—O characteristic peak (binding energy 286.3 eV) and the C-O-C characteristic peak (binding energy 287.7 eV). Compared with St, OSA-St displayed a new peak near 288.7 of the C1s spectra belonging to the carbon of C=O. This result indicated that the OSA groups were successfully grafted on starch(Gao et al., 2023). After oxidation, the increasing peak of C=O suggested that the starch had been successfully oxidized. Furthermore, as the oxidation time increased, the glucose ring structure in the starch bone started to oxidize. The formation of semiacetal structures by the carbonyl and hydroxyl groups as well as the cleavage of the carbon monomers would increase the content of C-O-C structures, leading to a decrease in the C=O and C—O peak areas of OSA-OSt-2 and an increase in the peak area of C-O-C. The C—C and C—H peak areas of OSA-OSt-4 have doubled, which indicated that the C—O bond was undergoing further oxidation to form C=O, leading to the formation of a larger number of C—C and C—H bonds.

As shown in Fig. 3D, the O1s narrow spectrum of samples exhibited one peak which represented C—O (532.6 eV). A new peak representing C=O (530.88 eV) was observed for OSA-St after esterification with octenyl succinic anhydride. In comparison to OSA-St, the C=O peak for OSA-OSt-2 showed the smallest value, which could be linked to the semi-acetal structure and the disruption of C—C bonding. The peak area of C=O also increased gradually with increasing ozone time, indicating that the starch had been successfully oxidized.

### 3.4 ^1^H-nuclear magnetic resonance analysis (^1^H NMR)

Fig. 3E illustrates the ^1^HNMR spectrums of St, OSA-St, and OSA-OSt (1–4). The chemical shift of ^1^H of St was assigned as follows: 5.10 ppm to H_1_ (internal, 1–4),5.05 ppm to H_1_ (non-reducing end), 4.92 ppm to H_1_(1–6), and 3.2–3.6 ppm to H_2_-H_6_, Additionally, the resonances at 5.51, 5.41, and 4.58 ppm were attributed to OH_2_, OH_3_, and OH_6_, respectively.

Compared with St, the OSA-St exhibited some additional resonances. Notably, the resonance of the terminal methyl protons (H_7_) was close to 0.86 ppm, H_8_-H_10_ to 1.22–1.29 ppm, H_11_ and H_14_–H_16_ to 1.93 ppm (Z. Zhang, Zhao, & Xiong, 2013). Furthermore, in the case of OSA-OSt-1, the peak areas of OH_2_ and OH_3_ decreased sharply (Table S2), indicating that oxidation had mainly occurred in C_2_ and C_3_. Dramatically, when the oxidation time exceeded 0.5 h, the variation in peak areas of OH_2_, OH_3,_ and OH_6_ exhibited no obvious trend, which might be due to the fracture of the glucose rings leading to the instability of the system. The three characteristic peaks corresponding to OH_2_, OH_3_, and OH_6_ in OSA-OSt-4 were absent, signifying the extreme structure change in OSA-St (Li et al., 2023). Fig.S1 illustrated a diagrammatic representation of potential structural alterations in OSA starch induced by ozone.

When starch is subjected to gaseous ozone, with ozone (O_3_) serving as a powerful oxidizer, it facilitates the oxidation of hydroxyl groups in OSA starch molecules, leading to the creation of aldehyde (CHO) and carboxylic (COOH) groups. The formation of C-O-C structures results from the interplay between the newly created carbonyl groups (C=O) and the hydroxyl groups that remain unreacted. Additionally, this mechanism triggers the breakdown of glycosidic ring formations in OSA starch, leading to the splitting of molecular chains and the creation of more fragments of lower molecular weight.

### 3.5 Thermal gravimetric analysis (TGA) and differential scanning calorimetry (DSC)

**Fig. 4:**
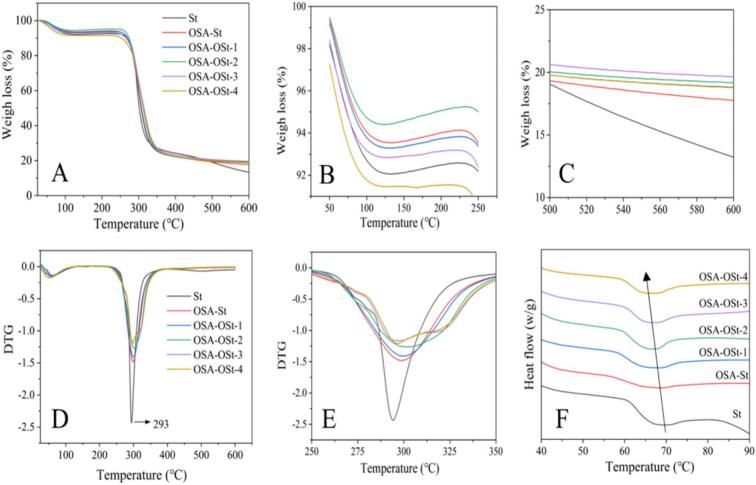
Thermal gravimetric analysis (TGA) (A-C), derivative thermogravimetric analysis (DGT) (D and E), and DSC (F) curves of St, OSA-St, and OSA-OSt with different oxidation degrees.

TGA (Thermal Gravimetric Analysis) and DTG (Derivative Thermogravimetric analysis) of St, OSA-St, and OSA-OSt with varying levels of oxidation are displayed in Fig. 4. There were three degradation stages observed on the TGA curves, where the first stage at 30–180 °C was corresponding to the water desorption, the second stage at 180–320 °C was related to the thermal degradation of the glucose ring in the starch chain, and the last stage at 320–600 °C suggested the degradation followed by the decarboxylation reaction and carbonization of the starch molecule chains (Meng et al., 2020). To distinguish the changes among those different starch samples, the TGA curves at 50–250 °C and 500–600 °C are presented in Fig. 4B and C in detail, respectively.

In Fig. 4B, compared to the St sample, OSA-St, OSA-OSt-1, and OSA-OSt-2 displayed a less loss percent in weight, because the process of modification utilized several hydroxyl groups enhanced the hydrophobicity of starch molecule, leading to a weak water immobilization. Further oxidation of the samples aggravated the molecular depolymerization that benefited the exposure of active hydroxyl groups, resulting in the increased connection between water and starch chains. Besides, it was found that the total weight loss percentage of the starch sample decreased from around 87% to 82% after esterification, the phenomenon was similar to the previous report that is related to the formation of residual sodium octenyl succinate salt(Xiao, Weng, Chen, & Xiao, 2019). As the oxidation time was increased from 0.5 to 2 h, the weight loss percentage was followed by decrease from approximately 87% to 80%. This was similar to several previously reported works,(Y.-R. Zhang, Wang, Zhao, & Wang, 2012; Zuo et al., 2017) in which the hemiacetal reaction during the oxidation was attributed to preventing further weight loss during the thermal degradation (Afinjuomo et al., 2020). From the DTG results, the OSA-St sample displayed a higher maximum weight-decomposing temperature (around 300 °C) than that of St (around 293 °C) but a lower decomposing rate, suggesting the promotion of OSA groups on starch thermal stability. Remarkably, a trend of increasing first and then decreasing about the maximum weight-decomposing temperature was noted with the increasing oxidation time. The change in the balance between oxidation-induced crosslinking and depolymerization could explain this phenomenon. If the oxidation time did not exceed 1 h, then the enhanced molecular interaction endowed the starch with enhanced thermal stability. Once the oxidation time exceeded 1 h, the breakage of the starch chain would lower the thermal stability consequently.(Castanha, Lima, Matta, Campanella, & Augusto, 2019; Hu et al., 2022; Li et al., 2023) The presence of a double peak on the curves of OSA-OSt-3 and OSA-OSt-4 also proved the molecular depolymerization.

The DSC curves of the samples with different durations of ozone treatment are shown in Fig. 4F. The decrease in the temperature of gelatinization indicated that the esterification and oxidation could accelerate the entrance of water into the starch granule during the heating process (Chapagai et al., 2021). This was in agreement with the previous report, where the low gelatinization temperature was mainly responsive to the changes in surface structure and crystallinity of starch(Oladebeye et al., 2013; Wang et al., 2022).

### 3.6 Rapid viscosity analysis (RVA)

The RVA equipment can measure how the apparent viscosity of a starch solution changes over time under different temperatures. The pasting parameters and curves of St, OSA-St, and OSA-OSt-1,2,3,4 samples are shown in Table 1 and Fig.S2.

**Table 1:** RVA parameters of St, OSA-St, and OSA-OSt with different oxidation degrees. Peak viscosity (PV), Breakdown (BD), Final viscosity (FV), Setback (SB), and Pasting temperature (PT).

Sample ID	Peak Visc (PV)	Breakdown (BD)	Final Visc (FV)	Setback (SB)	Pasting Temp (PT)
St	2786 ± 16.97^c^	1651 ± 8.49^d^	1414.5 ± 9.19^c^	279.5 ± 0.71^bc^	69.15 ± 0.57^a^
OSA-St	4385 ± 280.01^b^	2067 ± 1.41^b^	2670.5 ± 161.93^b^	352.5 ± 116.67^b^	62.75 ± 0.14^c^
OSA-OSt-1	6739.5 ± 306.18^a^	4718 ± 120.21^a^	3003 ± 86.27^a^	1031.5 ± 201.53^a^	65.675 ± 0.6^b^
OSA-OSt-2	4716 ± 22.63^b^	1878.5 ± 92.63^c^	3130 ± 53.74^a^	292.5 ± 16.26^bc^	69.05 ± 0.64^a^
OSA-OSt-3	2531.5 ± 28.99^c^	1077.5 ± 116.67^e^	1554 ± 89.1^c^	100 ± 1.41^cd^	69.38 ± 0.11^a^
OSA-OSt-4	1061 ± 128.69^d^	617 ± 52.33^f^	501 ± 89.1^d^	57 ± 12.73^d^	69.4 ± 0.07^a^

Note: tests were performed in triplicate. Mean ± standard deviation values in the same column for each sample followed by different letters are significantly different (*p* < 0.05).

As noted in Fig.S2, OSA-St showed a higher peak viscosity than that of the St, because the OSA modification caused particle swelling along with a minor disruption of the starch fraction in the granules. Moreover, 0.5 h of oxidation (OSA-OSt-1) resulted in the highest peak viscosity, which was attributed to partial cleavage of glycosidic bonds (Okekunle, Adebowale, Olu-Owolabi, & Lamprecht, 2020). Obviously, when the treating time exceeded 0.5 h, the peak viscosity of OSA-OSt decreased with the increasing time (Castanha et al., 2017). This result signified that moderate ozone treating time played a dominant role in weakening the molecular interaction (eg. hydrogen bond), enabling a high degree of swelling. In Table 1, it was also found that the values of breakdown, setback, and final viscosity of starch exhibited a similar trend to peak viscosity. These results proved that 0.5 h ozone treatment (OSA-OSt-1) was in favor of improving the dispersion stability of OSA-St. Furthermore, the changing trend of pasting temperature following the DSC result demonstrates the influence of ozone on the thermal behavior of OSA starch.

### 3.7 Micromorphology of starch-based emulsion

**Fig. 5:**
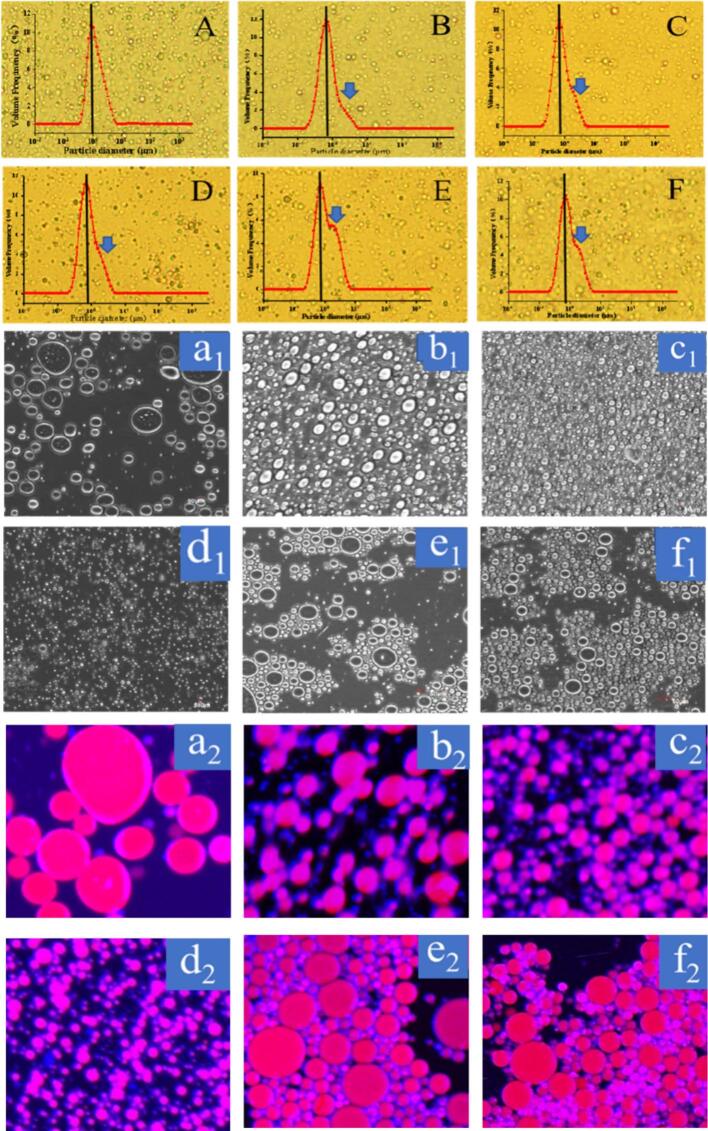
Optical micrographs of St, OSA-St, and OSA-OSt-1,2,3,4 (A-F) stabilized emulsions; and CLSM micrographs (brightfield microscopy (a_1_-f_1_) and multi-channel laser confocal microscopy (a_2_-f_2_)) of St, OSA-St, and OSA-OSt-1,2,3,4 stabilized emulsions,

Different starch samples were applied to prepare the emulsion and the optical and CLSM micrographs are depicted in Fig. 5. It was noted that every sample exhibited the characteristics of a standard oil-in-water (O/W) emulsion. Compared with St emulsion, the esterification that promoted the starch hydrophobicity homogenized the size of emulsion droplets. Consequently, the OSA-St emulsion exhibited a right-shift size distribution (No & Shin, 2019). Following ozone treatment for 0.5 and 1 h, a progressive reduction in droplet particle size was observed within the emulsion system, as illustrated in Fig. 5C and D. This was attributed to the strengthened surface repulsive force and dense hydrophobic lay. However, in Fig. 5C and D, the intensity pointed out with the blue arrow increased with increasing time. Here, this could be attributed to the existence of weak interaction. Similarly, a longer ozone processing time made the droplets agglomerative. By combining the above-mentioned thermal behavior, the ozone-induced aggregation or increasing size was related to the leaching or extending starch chains which promoted the hydrogen bonding interaction among the droplet stabilized with starch molecules.

### 3.8 Emulsion temperature dependence analysis

Thermal stability is of great importance for the application of emulsions in an environment under high temperatures(Liu et al., 2023). Since esterification and oxidation have been proven to significantly alter the molecular state, the emulsion characteristics of different emulsion samples under heating treatment must be taken into consideration. As a comparison to normal temperature, the size of the freeze-thaw sample was also measured.

**Fig. 6:**
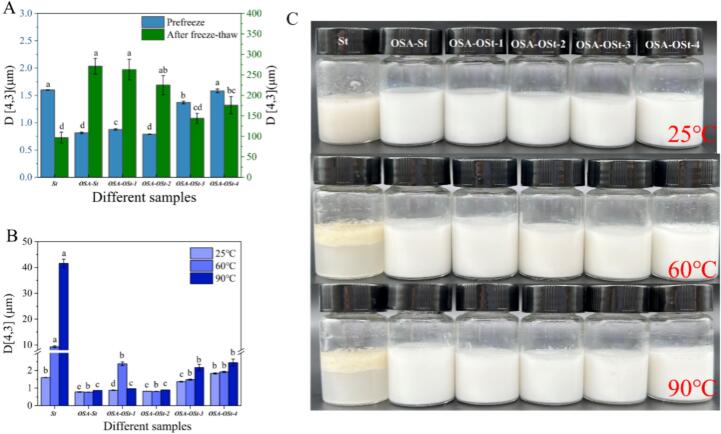
Distribution of different emulsions' particle sizes following heat treatment, and the emulsion pictures.

In Fig. 6A, it was found that the freeze-thaw increased the size of droplets in all starch-based emulsion samples. This was attributed to the weak flocculation or aggregation of the emulsion droplets due to the strengthening intermolecular interactions of gelatinized starch at low temperatures (Asiri, Ulbrich, & Flöter, 2022). The modified starch showed larger emulsion droplets, especially for the OSA-St and OSA-St-1 around 270 μm and 265 μm, respectively, which might be related to the higher pasting viscosity that suggested more complex molecular entanglement. Moreover, the ozone treatment for a relatively long time would further disorder the molecular, and promote the electric repulsive force (Castanha, Lima, et al., 2019). As a result, the decreasing particle size appeared after 2 and 3 h treatment.

We investigated the average particle size of different starch-based emulsions after the heating treatment at 25, 60, and 90 °C (Fig. 6B-C). For the St, the heating treatment resulted in the largest growth in droplet size. It was also noted that the droplet in the modified starch-based emulsion exhibited no significant growth in size. This result suggested the great thermal stability of the emulsion products. When the ozone treatment time exceeded 1 h, the increasing droplet size could be attributed to the leaching short starch chains.

### 3.9 Dynamical rheological behavior of emulsion

**Fig. 7:**
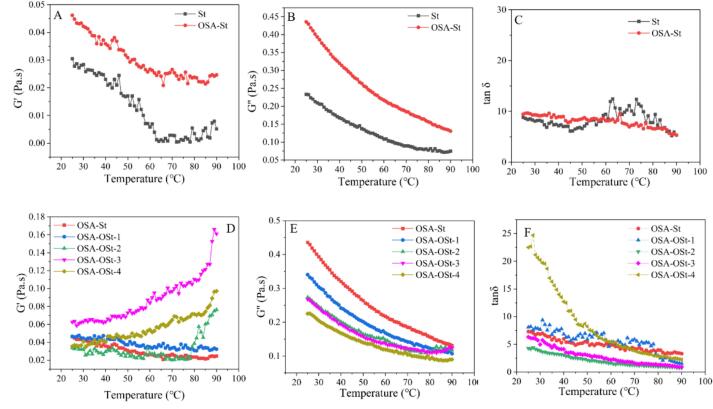
Storage modulus (G'), loss modulus (G′’), and tanδof St, OSA-St, and OSA-OSt-1, 2, 3, 4 emulsions as a function of temperature.

Dynamic temperature rheology is an efficient method to investigate the inner structure of starch-based emulsion systems. The alteration in magnitudes of storage modulus (G') and loss modulus (G′’) during the heating process could indirectly reflect how starch locating at the O/W interface affected the emulsion stability under different temperatures. As illustrated in Fig. 7A, the moduli of all starch-based emulsions displayed high-temperature dependence, and the G' of both St and OSA-St emulsion decreased as the temperature increased from 25 to 65 °C, reaching a minimum value at 65 and 90 °C, respectively. This was due to the accelerated movement of water molecules under high-temperature conditions, which limited the hydrogen bonding interaction of starch(Seo, Kang, Lee, Lee, & Chang, 2018). After oxidation, when treating time did not exceed 1 h, the relative low G' of OSA-St, OSA-OSt-1, and OSA-OSt-2 indicated the limited molecular interaction in the temperature range of 25–75 °C. When oxidation time reached 0.5 h, the higher G' of OSA-OSt-1 emulsion compared to that of OSA-St emulsion was probably due to the formation of weak-gel structure in the presence of extending starch chains. However, 1 h of oxidation would enhance the electric repulsive force between the droplets, leading the better dispersion but lower gel strength. With a further increase in treatment time, the leaching starch filled in the emulsion system played a role as the physical crosslinking agent to reinforce the gel structure, while the various balance between the repulsive force and crosslinking interaction resulted in the fluctuating elasticity of the emulsion system (Fig. 7D).

A decrease in the G′’ with the increase in temperature was observed in Fig. 7B and E. Obviously, the OSA esterification enabled the decreasing G′’ of OSA-St emulsion, suggesting the higher viscosity. It was also found that the G′’ of emulsion decreased with the increasing oxidation time, and such a gradient change argued that oxidation was not in favor of the viscous behavior of starch-based emulsion. This result was due to the dominant effect of molecular depolymerization and electrostatic interactions on the strength of the emulsion gel structure. Moreover, we also calculated the tan δ to evaluate the fluid behavior of the emulsion. In Fig. 7C and F, the tan δ of both St and OSA-St emulsions were <1, demonstrating the liquid-like state. When ozone treatment was performed, the OSA-OSt-1, 2, 3, and 4 emulsions showed different sol-gel behaviors. For the OSA-OSt-1 sample, the tan δ values were a little higher than that of the OSA-St emulsion but still <1 in the whole temperature range, suggesting that the main 0.5 h treatment on the emulsion structure was to restrict the molecular interaction. When ozone treatment time was increased to 1 h, the gel-like behavior of the emulsion was enhanced as the formation of a weak-gel structure, resulting in the reduced tan δ. Moreover, OSA-OSt-3 and OSA-OSt-4 exhibited the increasing tan δ, signifying that the excessive processing time of ozone could promote the liquid-like behavior of emulsion.

## 4 Conclusions

In this work, ozone was used to post-modify OSA starch and the emulsion formed with this dual-modified starch had improved thermal stability. It was found that due to oxidation disrupting starch structure leading to an increase in leached molecular chains, the swelling power and water-holding capacity were increased, while solubility decreased. Moreover, oxidation damaged the structure of starch and enhanced molecular interactions, and the dual-modified starch displayed enhanced thermal properties and high peak viscosity. Furthermore, the role of the leached oxidized starch was cross-linking, and the balance between crosslinking and electric charge caused the emulsion to be highly thermally stable. Overall, OSA starch and its emulsion can be endowed with superior thermal stability through the use of ozone for post-modification.

## CRediT authorship contribution statement

**Meng Du:** Writing – original draft, Validation, Software, Formal analysis, Data curation. **Zia-ud Din:** Writing – review & editing, Visualization. **Xinya Liu:** Writing – review & editing. **Xi Chen:** Writing – review & editing, Methodology, Funding acquisition, Conceptualization. **Yuehui Wang:** Resources. **Kun Zhuang:** Resources. **Lijie Zhu:** Formal analysis, Data curation. **Wenping Ding:** Supervision, Project administration, Funding acquisition.

## Declaration of competing interest

We declare that no conflict of interest exists in the submission of this manuscript entitled “Ozone induced structural variation in OSA waxy rice starch: Effects on the thermal behavior of starch and its stabilized Pickering emulsion”, and the manuscript is approved by all authors including Meng Du ^1^, ^2^, Lei Chen ^1, 2^*, Zia-ud Din ^3^, Xinya Liu ^2^, Xi Chen ^1, 2^, Yuehui Wang ^1^, Kun Zhuang ^1, 2^, Lijie Zhu ^1, 2^, Wenping Ding ^1, 2⁎^. I want to declare on behalf of my co-authors that the work described is original research that has not been published previously and is not under consideration for publication elsewhere, in whole or in part. All the authors listed have approved the manuscript that is enclosed.

## Data Availability

No data was used for the research described in the article.

## Appendix A Supplementary data

Supplementary material for the structural and RVA characterization of St, OSA-St, and OSA-OSt with different oxidation degrees, and the possible reaction mechanism Image 1

## References

[bb0005] Afinjuomo F., Fouladian P., Barclay T.G., Song Y., Petrovsky N., Garg S. (2020). Influence of oxidation degree on the physicochemical properties of oxidized inulin. Polymers.

[bb0010] Asiri S.A., Ulbrich M., Flöter E. (2022). Effect of pre-swelling and freezing/thawing cycles on the structure of molecular, morphological, and functional properties of potato starch. Journal of Food Biochemistry.

[bb0015] Boetje L., Lan X.H., van Dijken J., Woortman A.J.J., Popken T., Polhuis M., Loos K. (2023). Starch ester film properties: The role of the casting temperature and starch its molecular weight and amylose content. Carbohydrate Polymers.

[bb0020] Butt N.A., Ali T.M., Hasnain A. (2019). Rice starch citrates and lactates: A comparative study on hot water and cold water swelling starches. International Journal of Biological Macromolecules.

[bb0030] Castanha N., da Matta M.D., Augusto P.E.D. (2017). Potato starch modification using the ozone technology. Food Hydrocolloids.

[bb0035] Castanha N., Lima D.C., Matta M.D., Campanella O.H., Augusto P.E.D. (2019). Combining ozone and ultrasound technologies to modify maize starch. International Journal of Biological Macromolecules.

[bb0040] Castanha N., Miano A.C., Jones O.G., Reuhs B.L., Campanella O.H., Augusto P.E.D. (2020). Starch modification by ozone: Correlating molecular structure and gel properties in different starch sources. Food Hydrocolloids.

[bb0050] Castanha N., Santos D.N.E., Cunha R.L., Augusto P.E.D. (2019). Properties and possible applications of ozone-modified potato starch. Food Research International.

[bb0055] Chan H.T., Bhat R., Karim A.A. (2009). Physicochemical and functional properties of ozone-oxidized starch. Journal of Agricultural and Food Chemistry.

[bb0060] Chan H.-T., Leh C.P., Bhat R., Senan C., Williams P.A., Karim A.A. (2011). Molecular structure, rheological and thermal characteristics of ozone-oxidized starch. Food Chemistry.

[bb0065] Chang S.Y., Dong S.P., Tech Assoc Graph A., Tech Assoc Graph A., Tech Assoc Graph A., Tech Assoc Graph A. (1997). 49th Annual Technical Conference of the Technical-Association-of-the-Graphic-Arts on Disseminating Graphic Arts Research Internationally Since 1948 (TAGA 97).

[bb0070] Chapagai M.K., Fletcher B., Witt T., Dhital S., Flanagan B.M., Gidley M.J. (2021). Multiple length scale structure-property relationships of wheat starch oxidized by sodium hypochlorite or hydrogen peroxide. Carbohydrate Polymer Technologies and Applications.

[bb0075] Du M., Chen L., Din Z.U., Zhan F.C., Chen X., Wang Y.H., Ding W.P. (2023). Structure and surface properties of ozone-conjugated octenyl succinic anhydride modified waxy rice starch: Towards high-stable Pickering emulsion. International Journal of Biological Macromolecules.

[bb0080] Feng Y.N., Zhang B., Fu X., Huang Q. (2022). Starch-lauric acid complex-stabilised Pickering emulsion gels enhance the thermo-oxidative resistance of flaxseed oil. Carbohydrate Polymers.

[bb0085] Fonseca L.M., Gonçalves J.R., el Halal S.L.M., Pinto V.Z., Dias A.R.G., Jacques A.C., Zavareze E.D. (2015). Oxidation of potato starch with different sodium hypochlorite concentrations and its effect on biodegradable films. LWT- Food Science and Technology.

[bb0090] Gao X., Du J., Cheng L., Li Z., Li C., Ban X., Hong Y. (2023). Fabrication of octenyl succinic anhydride starch grafted with folic acid and its loading potential for doxorubicin hydrochloride. International Journal of Biological Macromolecules.

[bb0095] Ge X., Duan H., Zhou Y., Zhou S., Shen H., Liang W., Yan W. (2023). Investigating the effects of pre- and post-electron beam treatment on the multiscale structure and physicochemical properties of dry-heated buckwheat starch. International Journal of Biological Macromolecules.

[bb0100] Ge Y., Shi Y., Wu Y., Wei C., Cao L. (2023). Preparation, structure, and in-vitro hypoglycemic potential of debranched millet starch-fatty acid composite resistant starch. Food Chemistry: X.

[bb0105] Handarini K., Hamdani J.S., Cahyana Y., Setiasih I.S. (2020). Gaseous ozonation at low concentration modifies functional, pasting, and thermal properties of arrowroot starch (Maranta arundinaceae). Starch-Starke.

[bb0110] He Y.L., Ye F.Y., Tao J.M., Zhang Z.H., Zhao G.H. (2023). Ozone exposure tunes the physicochemical properties of sweet potato starch by modifying its molecular structure. International Journal of Biological Macromolecules.

[bb0115] Hsieh C.-F., Liu W., Whaley J.K., Shi Y.-C. (2019). Structure and functional properties of waxy starches. Food Hydrocolloids.

[bb0120] Hu J., Li X., Cheng Z., Fan X., Ma Z., Hu X., Xing Y. (2022). Modified tartary buckwheat (Fagopyrum tataricum Gaertn.) starch by gaseous ozone: Structural, physicochemical and in vitro digestible properties. Food Hydrocolloids.

[bb0130] Huang X., Chen L., Liu Y. (2024). Effects of ultrasonic and ozone modification on the morphology, mechanical, thermal and barrier properties of corn starch films. Food Hydrocolloids.

[bb0135] Hui R., Qi-he C., Ming-liang F., Qiong X., Guo-qing H. (2009). Preparation and properties of octenyl succinic anhydride modified potato starch. Food Chemistry.

[bb0140] Kasprzak M., Wilde P., Hill S.E., Harding S.E., Ford R., Wolf B. (2019). Non-chemically modified waxy rice starch stabilised wow emulsions for salt reduction. Food & Function.

[bb0145] Klaochanpong N., Puncha-Arnon S., Uttapap D., Puttanlek C., Rungsardthong V. (2017). Octenyl succinylation of granular and debranched waxy starches and their application in low-fat salad dressing. Food Hydrocolloids.

[bb0150] Klein B., Vanier N.L., Moomand K., Pinto V.Z., Colussi R., Zavareze E.D., Dias A.R.G. (2014). Ozone oxidation of cassava starch in aqueous solution at different pH. Food Chemistry.

[bb0155] Laovachirasuwan P., Peerapattana J., Srijesdaruk V., Chitropas P., Otsuka M. (2010). The physicochemical properties of a spray dried glutinous rice starch biopolymer. Colloids and Surfaces. B, Biointerfaces.

[bb0160] Li G.T., Chen J.T., Zhu F. (2023). Comparative study of rheological properties and Pickering emulsion stabilizing capacity of nonenyl succinic anhydride and octenyl succinic anhydride modified amaranth starches. International Journal of Biological Macromolecules.

[bb0170] Li J., Du M., Din Z.-U., Xu P., Chen L., Chen X., Ding W. (2023). Multi-scale structure characterization of ozone oxidized waxy rice starch. Carbohydrate Polymers.

[bb0175] Li Y., Wang J.-H., Wang E.-C., Tang Z.-S., Han Y., Luo X.-E., Han Z. (2023). The microstructure and thermal properties of pulsed electric field pretreated oxidized starch. International Journal of Biological Macromolecules.

[bb0180] Liu Y., Tan Z., Huang Y., Liu J., Xu X., Zhu B., Dong X. (2023). pH-shift strategy improving the thermal stability and oxidation stability of rice starch/casein-based high internal phase emulsions for the application in fish cake. Food Chemistry: X.

[bb0185] Lv Q.-Q., Li G.-Y., Xie Q.-T., Zhang B., Li X.-M., Pan Y., Chen H.-Q. (2018). Evaluation studies on the combined effect of hydrothermal treatment and octenyl succinylation on the physic-chemical, structural and digestibility characteristics of sweet potato starch. Food Chemistry.

[bb0190] Meng R., Wu Z., Xie H.-Q., Xu G.-X., Cheng J.-S., Zhang B. (2020). Preparation, characterization, and encapsulation capability of the hydrogel cross-linked by esterified tapioca starch. International Journal of Biological Macromolecules.

[bb0195] Mu M., Karthik P., Chen J., Holmes M., Ettelaie R. (2021). Effect of amylose and amylopectin content on the colloidal behaviour of emulsions stabilised by OSA-modified starch. Food Hydrocolloids.

[bb0200] No J., Shin M. (2019). Preparation and characteristics of octenyl succinic anhydride-modified partial waxy rice starches and encapsulated paprika pigment powder. Food Chemistry.

[bb0205] Okekunle M.O., Adebowale K.O., Olu-Owolabi B.I., Lamprecht A. (2020). Physicochemical, morphological and thermal properties of oxidized starches from Lima bean (Phaseolus lunatus). Scientific African.

[bb0210] Oladebeye A.O., Oshodi A.A., Amoo I.A., Abd Karim A. (2013). Functional, thermal and molecular behaviours of ozone-oxidised cocoyam and yam starches. Food Chemistry.

[bb0215] Ovando-Martinez M., Whitney K., Ozsisli B., Simsek S. (2017). Physicochemical properties of octenyl succinic esters of cereal, tuber and root starches. Journal of Food Processing and Preservation.

[bb0220] Phan T.D., Debeaufort F., Luu D., Voilley A. (2005). Functional properties of edible agar-based and starch-based films for food quality preservation. Journal of Agricultural and Food Chemistry.

[bb0225] Pranoto Y., Paramita B.L., Cahyanto M.N., Benjakul S. (2021). Properties of ozone-oxidized tapioca starch and its use in coating of fried peanuts. Molecules.

[bb0230] Riyajan S.A., Poolyarat N. (2023). Cassava starch with ozone amendment and its blend: Fabrication and properties for fruit packaging application. Industrial Crops and Products.

[bb0235] Sandhu H.P.S., Manthey F.A., Simsek S. (2012). Ozone gas affects physical and chemical properties of wheat (<i>*Triticum aestivum*</i> L.) starch. Carbohydrate Polymers.

[bb0240] Seo S.Y., Kang Y.-R., Lee Y.-K., Lee J.H., Chang Y.H. (2018). Physicochemical, molecular, emulsifying and rheological characterizations of sage (Salvia splendens) seed gum. International Journal of Biological Macromolecules.

[bb0245] Shin S.I., Kim H.J., Ha H.J., Lee S.H., Moon T.W. (2005). Effect of hydrothermal treatment on formation and structural characteristics of slowly digestible non-pasted granular sweet potato starch. Starch-Starke.

[bb0250] Shweta Kumar Y., Saxena D.C. (2021). Valorization of unpopped foxnut starch in stabilizing Pickering emulsion using OSA modification. International Journal of Biological Macromolecules.

[bb0255] Simonin H., Guyon C., Orlowska M., de Lamballerie M., Le-Bail A. (2011). Gelatinization of waxy starches under high pressure as influenced by pH and osmolarity: Gelatinization kinetics, final structure and pasting properties. LWT- Food Science and Technology.

[bb0260] Siroha A.K., Bangar S.P., Sandhu K.S., Lorenzo J.M., Trif M. (2022). Octenyl succinic anhydride modified pearl millet starches: An approach for development of films/coatings. Polymers.

[bb0265] van Soest J.J.G., Tournois H., de Wit D., Vliegenthart J.F.G. (1995). Short-range structure in (partially) crystalline potato starch determined with attenuated total reflectance Fourier-transform IR spectroscopy. Carbohydrate Research.

[bb0270] Teixeira B.S., del Mastro N.L. (2023). Effects of electron beam irradiation on ozone-modified potato starch film. Radiation Physics and Chemistry.

[bb0275] Vandeputte G.E., Vermeylen R., Geeroms J., Delcour J.A. (2003). Rice starches. I. Structural aspects provide insight into crystallinity characteristics and gelatinisation behaviour of granular starch. Journal of Cereal Science.

[bb0280] Wang L., Li X., Gao F., Liu S., Wu Y., Liu Y., Zhang D. (2022). Effects of jet milling pretreatment and esterification with octenyl succinic anhydride on physicochemical properties of corn starch. Foods.

[bb0285] Wang Q., Tang Z., Li Z., Luan Y., Gu C., Liu R., Wu M. (2023). Effects of octenyl succinylation on the properties of starches with distinct crystalline types and their Pickering emulsions. International Journal of Biological Macromolecules.

[bb0290] Wang W.N., Liu C., Zhang H.R., Zhu X.Q., Wang L.Q., Zhang N., Yu D.Y. (2022). Properties of OSA-modified starch and emulsion prepared with different materials: Glutinous rice starch, japonica rice starch, and indica rice starch. Food Research International.

[bb0295] Xiao Q., Weng H., Chen G., Xiao A. (2019). Preparation and characterization of octenyl succinic anhydride modified agarose derivative. Food Chemistry.

[bb0300] Yulianingsih R., Gohtani S. (2019). Dispersion characteristics of pregelatinized waxy rice starch and its performance as an emulsifier for oil-in-water emulsions: Effect of gelatinization temperature and starch concentration. Food Hydrocolloids.

[bb0305] Zhang M., Xu Z., Zhang Q., Dan Z., Fu H., Yao W. (2023). Properties and potential application of ozone-oxidized starch for enhanced reverse flotation of fine hematite. Minerals Engineering.

[bb0310] Zhang Y., Dai Y., Hou H., Li X., Dong H., Wang W., Zhang H. (2020). Ultrasound-assisted preparation of octenyl succinic anhydride modified starch and its influence mechanism on the quality. Food Chemistry: X.

[bb0315] Zhang Y.-R., Wang X.-L., Zhao G.-M., Wang Y.-Z. (2012). Preparation and properties of oxidized starch with high degree of oxidation. Carbohydrate Polymers.

[bb0320] Zhang Z., Zhao S., Xiong S. (2013). Molecular properties of octenyl succinic esters of mechanically activated Indica rice starch. Starch-Starke.

[bb0325] Zuo Y., Liu W., Xiao J., Zhao X., Zhu Y., Wu Y. (2017). Preparation and characterization of dialdehyde starch by one-step acid hydrolysis and oxidation. International Journal of Biological Macromolecules.

