# Supplementary material for: Ozone induced structural variation in OSA waxy rice starch: Effects on the thermal behavior of starch and its stabilized pickering emulsion
Source: Food Chem X. 2024 Jul 27;23:101701. doi: 10.1016/j.fochx.2024.101701 (PMC11342896; doi:10.1016/j.fochx.2024.101701)
Supplement: Supplementary file 1 — Supplementary material for the structural and RVA characterization of St, OSA-St, and OSA-OSt with different oxidation degrees, and the possible reaction mechanism [file mmc1.docx]

**Ozone induced structural variation in OSA waxy rice starch: Effects on the thermal behavior of starch and its stabilized Pickering emulsion**

Meng Du ^1, 2^, Lei Chen ^1, 2^*,Zia-ud Din ^3^, Xinya Liu ^2^, Xi Chen ^1, 2^, Yuehui Wang^1^, Kun Zhuang^1, 2^,Lijie Zhu ^1, 2^, Wenping Ding ^1, 2^*

1. Key Laboratory for Deep Processing of Major Grain and Oil, Ministry of Education, Hubei Key Laboratory for Processing and Transformation of Agricultural Products, Wuhan Polytechnic University, Wuhan 430023, P. R. China
2. School of Food Science and Engineering, Wuhan Polytechnic University, Wuhan 430023, P. R. China
3. Department of Microbiology and Biotechnology, Atta ur Rahman School of Applied Biosciences (ASAB), National University of Sciences and Technology (NUST), H-12 Islamabad 44000, Pakistan.

*** Corresponding author:**

* Corresponding author: chenleiy@whpu.edu.cn, chenleij921@sina.com (Lei Chen); whdingwp@163.com (Wenpin Ding)

#These authors contributed equally to this work.

**Table S1. Structural characteristics determined by FT–IR.**

| Different samples | 1050 cm^-1^/1021 cm^-1^ (DO) | 1021 cm^-1^/995cm^-1^ (DD) |
| --- | --- | --- |
| St | 1.64 | 0.97 |
| OSA-St | 1.58 | 1.01 |
| OSA-OSt-1 | 1.40 | 1.22 |
| OSA-OSt-2 | 1.43 | 0.85 |
| OSA-OSt-3 | 1.44 | 1.12 |
| OSA-OSt-4 | 1.41 | 1.27 |

**Table S2. Peak values of OH_2_, OH_3_, and OH_6_ for different samples**

| Sample ID | H_7_ | -OH_2_ | -OH_3_ | -OH_6_ |
| --- | --- | --- | --- | --- |
| OSA-St | 1 | 25.59 | 2.12 | 10.43 |
| OSA-OSt-1 | 1 | 14.36 | 0.66 | 8.86 |
| OSA-OSt-2 | 1 | 29.44 | 5.10 | 12.88 |
| OSA-OSt-3 | 1 | 15.58 | 18.41 | 11.68 |
| OSA-OSt-4 | 1 | ---- | ---- | ---- |


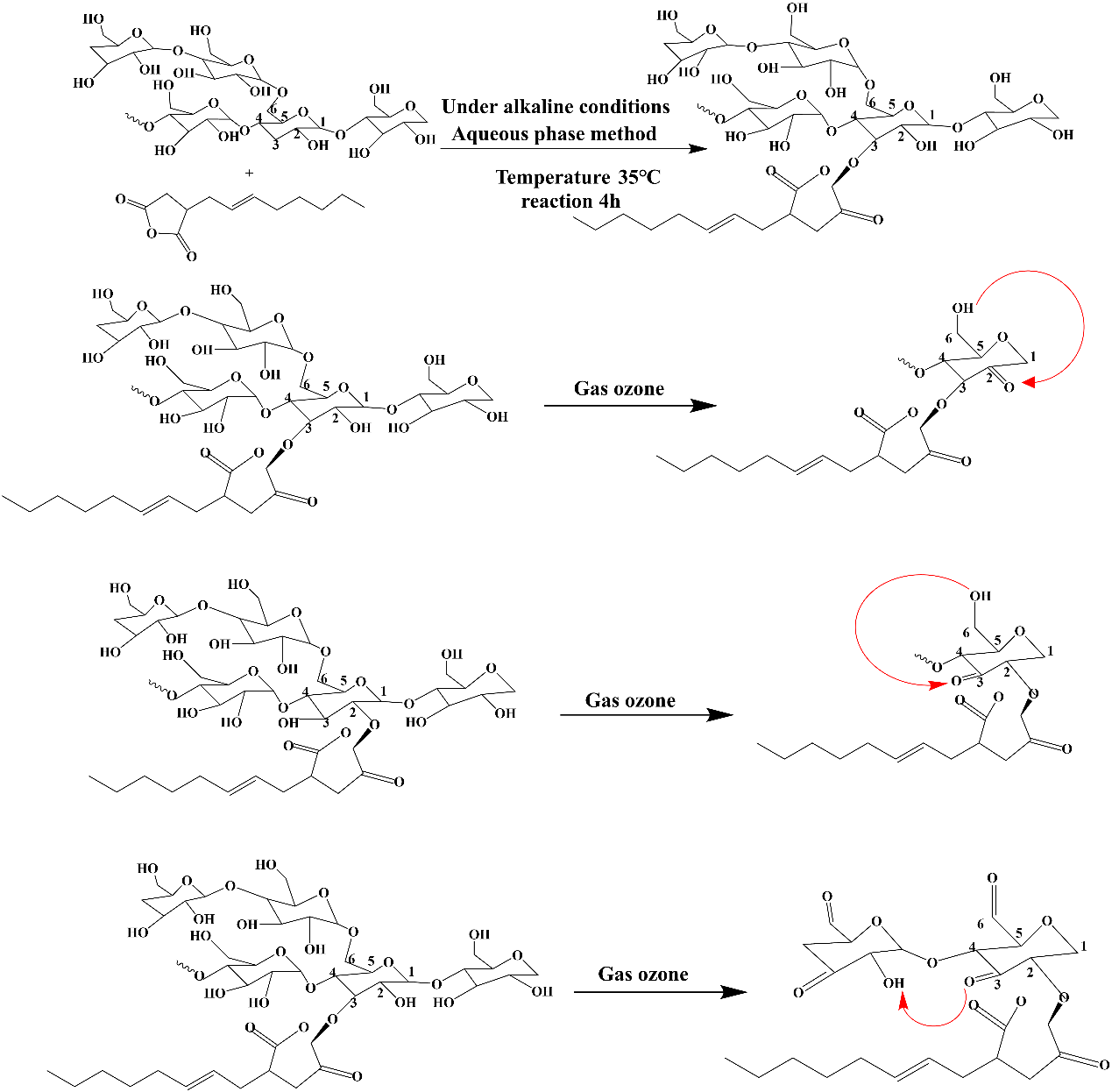


Fig. S1. Possible structural changes in OSA starch induced by ozone


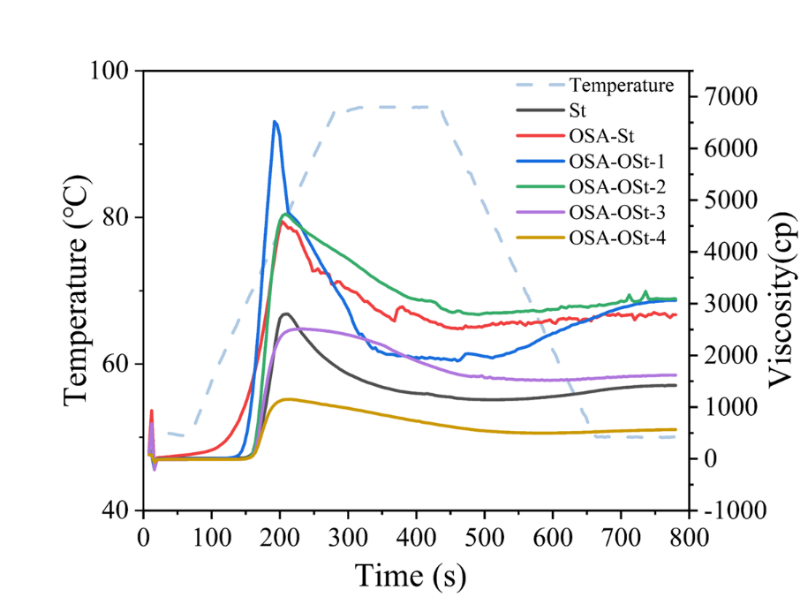


Fig. S2. The RVA curves of St, OSA-St, and OSA-OSt with different oxidation degrees

As noted in Fig. S2, OSA-St showed a higher viscosity peak than that of the St, because the OSA modification caused particle swelling along with a minor disruption of the starch fraction in the granules. Moreover, 0.5 h of oxidation (OSA-OSt-1) resulted in the highest peak viscosity, which was directly linked to high molecular flexibility. When the treating time exceeded 0.5 h, the peak viscosity of OSA-OSt decreased with the increasing time. This result signified that moderate ozone treating time plays a dominant role in weakening the molecular interaction (eg. hydrogen bond), enabling a high degree of swelling.
